# Coexistence of Chronic Myelomonocytic Leukemia and Ulcerative Colitis With Rapid Progression to Acute Myelomonocytic Leukemia: A Case Report

**DOI:** 10.7759/cureus.22422

**Published:** 2022-02-21

**Authors:** Lauren J Pelkey, David M Graham, Michael H Zakem, Michelle M Muza-Moons

**Affiliations:** 1 Medicine, Ascension Genesys Hospital, Grand Blanc, USA; 2 Pathology, University of Michigan Health-West, Wyoming, USA; 3 Oncology/Hematology, University of Michigan Health-West, Wyoming, USA; 4 Gastroenterology, University of Michigan Health-West, Wyoming, USA

**Keywords:** acute myelomonocytic leukemia, covid-19, ulcerative colitis, systemic inflammatory and autoimmune disease, chronic myelomonocytic leukemia

## Abstract

Chronic myelomonocytic leukemia (CMML) is a clonal myeloid neoplasm characterized by sustained peripheral blood monocytosis and variable dyspoiesis. We present a case of a 64-year-old male who presented with severe non-bloody diarrhea, peripheral blood neutrophilia, and monocytosis. He was diagnosed with myeloproliferative CMML type 0 and ulcerative colitis (UC). Next-generation DNA sequencing of a bone marrow sample demonstrated mutations of the TET2, ASXL1, NRAS, and SRSF2 genes along with low-level JAK2^V617F mutation. Both TET2 and SRSF2 mutations are associated with systemic inflammatory and autoimmune disease (SIAD), which includes UC. The patient's UC was managed successfully with vedolizumab infusions. The patient’s concurrent CMML was monitored with a “wait and watch” approach. After five months, the patient asymptomatically tested positive for coronavirus disease 2019 (COVID-19). Seven months after his diagnosis of CMML, the patient presented in severe respiratory distress with acute left upper quadrant pain, splenomegaly, and multiorgan failure. A peripheral blood smear demonstrated marked leukocytosis (283 x 10^9 /L) with 39% blasts/promonocytes without Auer rods. The patient was diagnosed with acute myeloid leukemia with myelomonocytic features (AMML). In this report, we discuss the diagnosis of combined CMML and SIAD, mechanisms of immunoregulatory dysfunction that have been suggested to result in CMML progression, and the clinicopathologic significance of the patient’s molecular abnormalities.

## Introduction

Chronic myelomonocytic leukemia (CMML) is a clonal myeloid neoplasm that can have features of both myeloproliferative neoplasm and myelodysplastic syndrome. CMML is divided into three categories (CMML-0, CMML-1, and CMML-2) based on the number of blasts in the peripheral blood and bone marrow and the presence or absence of Auer rods. Yearly incidence is estimated to be 0.3 cases/100,000 persons [1]. The median age at diagnosis is 73 years, with a male predominance (~2.5:1) [2]. CMML is thought to be due to an accumulation of age-related mutations in hematopoietic stem cells and may arise secondary to clonal hematopoiesis of indeterminate potential (CHIP) or in those with past exposure to chemotherapy/ionizing radiation. Karyotypic abnormalities occur in only a minority (27%) of patients [3]. Gene mutations occur in over 90% of cases and involve a variety of sites, including but not limited to TET2, SRSF2, ASXL1, RAS, and DNMT3A, though no mutations are specific for CMML [4]. Patients tend to present with symptoms related to myeloproliferation (hepatosplenomegaly, leukocytosis, night sweats, weight loss, and fatigue) or myelodysplasia (thrombocytopenia, leukopenia, and anemia). Diagnosis is based largely on excluding other myeloproliferative and myelodysplastic neoplasms and on a combination of peripheral blood (persistent monocytosis >1 x 10^9/L), bone marrow, and genetic abnormalities.

Due to the clinicopathologic heterogeneity of CMML, therapy is individualized, ranging from a “wait and watch” approach with minor disease severity, to erythropoiesis-stimulating agents in cases of anemia, to cytoreductive treatment (hydroxyurea) in cases of proliferative disease, to hypomethylating agents (5-azacitidine, decitabine) in the most severe cases of CMML, especially in the setting of peripheral blood cytopenias [5]. Many risk stratification models exist, but no single model is considered superior. Poor prognostic indicators include hemoglobin less than 10 g/dL, high-risk karyotypes, and the presence of circulating blasts [2]. The only current curative treatment is allogeneic bone marrow transplantation, which requires high-dose chemotherapy that is clinically contraindicated in 90% of CMML patients [5]. Average survival time after diagnosis is about 20 months [6]. Death due to transformation to acute myeloid leukemia (AML) occurs in 15-29% of CMML patients over three to five years [2].

We present a case of a 64-year-old male presenting with non-bloody diarrhea, peripheral blood neutrophilia, and monocytosis who was diagnosed with CMML type 0 and ulcerative colitis (UC). UC is one of the systemic inflammatory and autoimmune diseases (SIAD) [7]. Patients with combined CMML and SIAD exhibit a unique clinical course and response to therapy in comparison to patients with CMML alone [8]. In this study, we discuss the underlying mechanisms that may explain the immunoregulatory dysfunction in our patient, with concurrent SIAD and rapid progression to acute myelomonocytic leukemia.

## Case presentation

**Figure 1:**
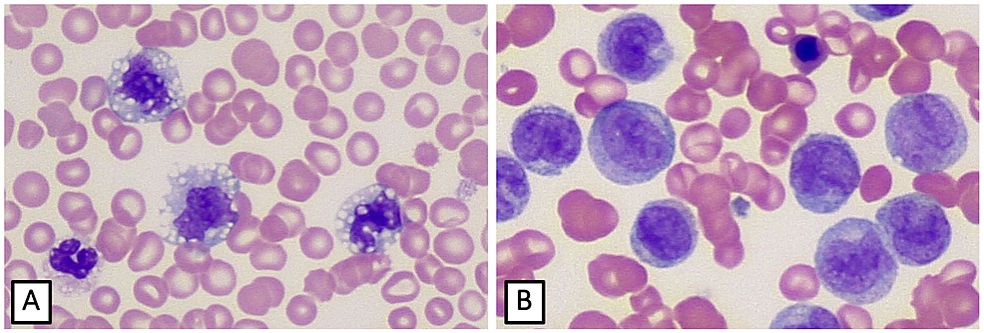
Wright stained peripheral blood smear before (A) and after (B) transformation to acute myelomonocytic leukemia (x400) (A) Initial presentation revealing a peripheral blood monocytosis. (B) Seven months after initial presentation demonstrated transformation, as evidenced by the high proportion of blasts, promonocytes, monocytes, and nucleated erythrocytes.

A 64-year-old Caucasian male presented with cough and dyspnea. The patient’s past medical history was significant for gastroesophageal reflux, type II diabetes, hypertension, Barrett esophagus, and chronic joint pain. His family history was negative for malignancy or systemic autoimmune or inflammatory disease. He received a presumptive diagnosis of pneumonia and was treated with azithromycin. Two weeks later, he presented to the ER with a several-day history of non-bloody diarrhea, night sweats, nausea, and fever (101^oF). Additional history documented a recent 15-20 pound weight loss. Physical examination was unremarkable except for diffuse, mild abdominal tenderness. Initial laboratory tests demonstrated elevated C-reactive peptide (120.2 mg/L). A peripheral blood smear demonstrated leukocytosis (55.2 x 10^9 cells/L), thrombocytopenia (97,000/µL), and mild anemia (hemoglobin 12.2 g/dL). The peripheral blood differential demonstrated absolute (5 x 10^9 /L) and relative (13%) monocytosis, and absolute (~45 x 10^9 /L) and relative (80%) neutrophilia (Figure 1A). Imaging studies demonstrated mild splenomegaly and non-specific mesenteric lymphadenopathy. GI workup including sigmoidoscopy demonstrated the presence of continuous moderate to severe inflammation involving the rectum and sigmoid colon (Mayo score 2-3 colitis). Based on the endoscopy and biopsy results, the patient was diagnosed with ulcerative colitis.

A bone marrow biopsy and aspirate were performed. The bone marrow biopsy revealed hypercellular marrow (95% cellularity), with substantially increased granulocytes, monocytes, and variably sized, hypolobulated megakaryocytes without micromegakaryocytes. The Wright-stained bone marrow aspirate smear differential disclosed 1% blasts, 82% maturing granulocytes, 5% monocytes, 3% lymphocytes, 7% erythroid precursors, 0% eosinophils, 0% basophils, and 2% mature plasma cells. The myeloid to erythroid (M:E) ratio was increased at 11.9, reflecting both increased granulocytic cells and somewhat decreased erythroid precursors. Only subtle, mild dysgranulopoiesis was appreciated. A reticulin stain performed on the core biopsy demonstrated patchy, slightly increased reticulin fibers, findings consistent with prefibrotic primary myelofibrosis (MF-0) or early-stage primary myelofibrosis (MF-1). Prussian blue stain showed adequate stainable storage iron and no ring sideroblasts. Immunohistochemical stains performed on the core biopsy demonstrated less than 1% cluster of differentiation (CD)34+ myeloblasts, scattered atypical CD61+ megakaryocytes displaying minimal dysplasia, and labeling of 3% of cells for CD117 - an immature granulocyte marker.

Genetic abnormalities included a low-level JAK2^V617F mutation in the peripheral blood. Additionally, peripheral leukocytes were negative for p210 and p190 BCR-ABL1 transcripts, with no BCR-ABL Philadelphia chromosome present. Leukocytes exhibited a normal male karyotype (46, XY). Next-generation DNA sequencing disclosed mutations in the following genes: TET2, ASXL1, NRAS, and SRSF2.

The findings warranted classification of the neoplasm as CMML-0 due to the absence of elevated numbers of blasts in the peripheral blood and bone marrow. Further, it was categorized as CMML-myeloproliferative type, due to leukocytosis >13,000 cells/µL and the presence of minimal dysplasia.

The patient's acute presentation of non-bloody diarrhea and constitutional symptoms were attributed to the UC. These symptoms were treated with a four-week course of moderate steroids as well as vedolizumab (Entyvio) infusions. Due to being unable to attribute any specific symptoms to the CMML, management was with a "wait and watch" approach. 

On follow-up, no significant changes in cell counts were observed over the next six months. His UC symptoms resolved and he was noted to be having one to two formed stools per day. He tolerated the steroids and vedolizumab well with no reported side effects. After six months, the patient tested positive for COVID-19 by reverse transcription-polymerase chain reaction (RT-PCR) but did not experience any COVID-related symptoms. He was unvaccinated for COVID-19 due to the vaccine not being available at the time.

Seven months after the initial presentation, the patient presented to the ER with a three-week history of constitutional symptoms (night sweats, fatigue, and fever) along with a three-day history of blood-tinged sputum, cough, shortness of breath, acute left upper quadrant pain, and obstipation. Chest x-ray revealed mild peribronchial infiltrate. CT showed massive hepatosplenomegaly with splenic infarcts, diffuse adenopathy throughout the abdomen/pelvis, and gastric wall thickening. The peripheral blood smear showed marked leukocytosis (283 x 10^9/L), anemia (Hb: 7.6 g/dL), and thrombocytopenia (30,000/µL). The peripheral blood differential demonstrated 39% circulating blasts/promonocytes, 23% monocytes, 13% immature/dyspoietic granulocytes, 24% bands/segs, and 1% lymphocytes (Figure 1B). There were also six nucleated red blood cells (RBCs) per 100 white blood cells (WBCs). No Auer rods were seen. The patient was diagnosed with acute myeloid leukemia with features of acute myelomonocytic leukemia (AMML). Additional labs revealed elevated liver function tests (alanine aminotransferase {ALT}: 509 IU/L; aspartate aminotransferase {AST}: 353 IU/L), elevated blood urea nitrogen, and creatinine (BUN: 57 mg/dL; Cr: 2.88 mg/dL), elevated cardiac enzymes (hs-troponin: 70 ng/L), high alkaline phosphatase (509 IU/L) and metabolic acidosis with hypoxemic respiratory failure. Due to the low likelihood of remission of AMML, the patient was treated palliatively, including daily oral 4 mg dexamethasone, 1000 mg hydroxyurea, and hospice.

## Discussion

CMML was originally classified in 1982 by the French-American-British (FAB) system as a myelodysplastic syndrome [2]. In 1994, CMML was dichotomized into myelodysplastic (leukocyte count < 13,000 cells/µL) and myeloproliferative types (leukocyte count > 13,000 cells/µL), with the latter being associated with a worse prognosis and higher likelihood of transformation to AML [2]. Since 2001, CMML has been grouped within the umbrella of myelodysplastic/myeloproliferative neoplasms (MDS/MPN).

In 2008, CMML was subdivided based on the number of blasts in the blood and bone marrow and the presence or absence of Auer rods. CMML type 0 has less than 2% blasts in the peripheral blood and less than 5% blasts in the bone marrow. CMML type I has 2-4% blasts (promonocytes, monoblasts, myeloblasts) in the blood or 5-9% blasts in the bone marrow. CMML type 2 is associated with the worst prognosis and demonstrates 5-19% blasts in the blood, 10-19% blasts in the bone marrow, or the presence of Auer rods [2]. In 2016, the WHO revised their criteria for CMML, based on advances in the detection of unique biomarkers associated with some myeloid neoplasms [9]. The most recent proposed diagnostic criteria for CMML were determined by an international consensus group, in August 2018 (Table 1) [10]. Here, we discuss the criteria for the patient’s diagnosis of CMML, his clinical course, and the clinicopathologic significance of his mutational profile (Video in the Appendices).

**Table 1:** Diagnostic criteria for chronic myelomonocytic leukemia *Cases of classic MPN can be associated with monocytosis or they can develop it during the course of the disease. These cases may simulate CMML. In these rare instances, previously documented history of MPN excludes CMML, whereas the presence of MPN features in the bone marrow and/or of MPN-associated mutations (JAK2, CALR, or MPL) tend to support MPN with monocytosis rather than CMML. The diagnosis of CMML can be established when all of the prerequisite criteria (A) and either morphologic dysplasia (B) or one or more of the co-criteria (C) are fulfilled. MPN: myeloproliferative neoplasm(s); CML: chronic myeloid leukemia; PMF: primary myelofibrosis; PV: polycythemia vera; ET: essential thrombocythemia; PDGFRA/B: platelet-derived growth factor receptor alpha/beta; FGFR1: fibroblast growth factor receptor 1; PCM1-JAK2: pericentriolar material 1, Janus kinase 2; AML: acute myeloid leukemia; CMML: chronic myelomonocytic leukemia Source: Arber et al., 2016 [9] and Valent et al., 2019 [10].

(A) Prerequisite criteria (all must be fulfilled)
Persistent (3 months) absolute (>1 × 10^9 /L) and relative (>10%) peripheral blood monocytosis
Exclusion of classic MPN: BCR-ABL1+ CML, PMF, PV, or ET*
No evidence of PDGFRA, PDGFRB, or FGFR1 rearrangement or PCM1-JAK2 (should be specifically excluded in cases with eosinophilia)
<20% blasts in the blood and bone marrow and exclusion of AML
(B) Dysplasia
Dysplasia in >10% of all cells in one of the following lineages in the bone marrow smear: erythroid, neutrophilic, megakaryocytic
(C) Co-criteria (for patients fulfilling A but not B, and otherwise showing typical clinical features of CMML such as splenomegaly)
Karyotypic abnormalities found in CMML (+8, -Y, -7/7q-, 20q-, etc.)
Expected molecular abnormalities in CMML (TET2, SRSF2, ASXL1, etc.)
Immunohistochemistry or flow cytometry findings showing leukemic infiltration of CD14+ monocytes

The diagnosis of CMML is largely one of exclusion. In our patient, BCR-ABL1+ chronic myeloid leukemia was excluded by the absence of BCR-ABL1 rearrangement. The patient did not have a history of myeloproliferative neoplasm (MPN) or MPN-associated driver mutation. The patient did exhibit a low-level JAK2^V617F mutation, which does not exclude the diagnosis of CMML [10]. JAK2^V617F mutation is found in many chronic myeloid neoplasms. It is present in >95% of patients with polycythemia vera (PV) and slightly >50% of patients with essential thrombocythemia (ET) and primary myelofibrosis (PMF) [11]. The diagnosis of PV was excluded by the presence of anemia and high M:E ratio. ET was excluded by the presence of thrombocytopenia and the lack of significant fibrosis. The patient’s bone marrow did reveal a mild increase in reticulin. An early, hypercellular primary myelofibrosis with monocytosis was excluded; however, because these are associated with high allelic burden of JAK2^V617F, higher proportion of MF-2/MF-3 reticulin fibrosis, and clustered megakaryocytes with a high degree of histologic atypia. In contrast, CMML patients can demonstrate low levels of JAK2^V617F, MF-1 reticulin fibrosis, and hypolobulated, dysplastic megakaryocytes which were all present in our patient [12]. CMML patients with any degree of bone marrow fibrosis have been found to have a worse prognosis than those without [13].

Other entities that were considered included atypical BCR-ABL1^- CML and MDS/MPN-unclassifiable were excluded because of the relative and absolute monocytosis. MDS/MPN with ring sideroblasts was excluded because a Prussian blue stain failed to reveal ring sideroblasts in the bone marrow. Myeloid/lymphoid neoplasm with eosinophilia was excluded due to the absence of eosinophilia and lack of mutations in PDGFRA, PDGFRB, FGFR1, or PCM1-JAK2 gene rearrangement.

Molecular abnormalities

Mutations in CMML can be grouped according to mutations in epigenetic modification (ASXL1, TET2, IDH), DNA methylation (DNMT3A), cell signaling (JAK2^V617F, CBL, NRAS/KRAS, PTPN11, FLT3), spliceosome machinery mutations (SRSF2, SF3B1, U2AF1), transcription/nucleosome assembly mutations (RUNX1, SETBP1), and/or DNA damage repair mutations (PHF6, TP53) [4]. Our patient tested positive for TET2, SRSF2, ASXL1, NRAS, and JAK2^V617F mutations. TET2 mutations are the earliest and most common mutations in CMML, occurring in 60% of cases [4]. In myeloid cells, TET2 functions to inhibit IL-6 and resolve an inflammatory state [14]. Studies using mouse models have found TET2 loss to be associated with elevated cytokines/chemokines, resulting in increased susceptibility to colitis and IL-1β dependent atherosclerosis [14]. Additionally, both TET2 and SRSF2, genes involved in pre-mRNA splicing, are associated with systemic inflammatory and autoimmune disease (SIAD), an umbrella term that includes ulcerative colitis and infectious diseases, such as COVID-19 [7,15].

Oncogenic signaling mutations in JAK2^V617F and NRAS both result in granulocyte-macrophage colony-stimulating factor (GM-CSF) hypersensitivity, a finding in >90% of CMML cases [16]. These activating mutations confer a survival advantage to the mutant CMML cells, leading to uninhibited clonal expansion of granulomonocytic precursors in the bone marrow at the expense of erythroid precursors [17]. Additionally, JAK2^V617F mutations are found to induce increased reticulin fibers in the bone marrow, as found in our patient [18]. Finally, mutations in ASXL1, SRSF2, and the RAS pathway, which were found in our patient, are independently associated with the myeloproliferative subtype of CMML and a poor prognosis [3].

Ulcerative colitis and CMML

Ulcerative colitis (UC) has been reported to occur in 0.3% of CMML patients [19]. UC is thought to arise from immune system dysregulation due to an overactive immune response to gut microbiota with increased cytokine production (IL-1β, IL-6, tumor necrosis factor-alpha {TNFα}) resulting in skewed T-cell populations with a decreased number of circulating Tregs [20]. Treg number and function are dependent upon IL-2 signaling for proper transcriptional activity, which prevents the development of autoimmunity [21]. IL-2 is also necessary for natural killer (NK) cell function. NK cell function is decreased in both UC and CMML [22,23]. Increasing IL-2 signaling has been postulated as a possible therapy for UC/CMML patients since it may increase Treg populations and increase NK cell activity, leading to increased protection of colonic mucosa in UC and increased cytotoxicity directed to tumor cells in CMML [24,25]. Clinical trials are currently underway to see if IL-2 therapy results in biological response in UC/CMML patients [26-28].

Approximately 30% of CMML patients will have an associated systemic inflammatory and autoimmune disease (SIAD) [2]. In addition to inflammatory bowel disease, SIAD includes inflammatory arthritis, systemic vasculitis, autoimmune cytopenias, connective tissue diseases, and neutrophilic dermatoses [7]. Dual CMML and SIAD cases have a higher absolute neutrophil count, a lower proportion of blasts, and a higher frequency of TET2 and SRSF2 mutations in comparison to those with CMML alone [7]. Rates of transformation to AML and overall survival, however, are similar [8].

COVID-19 infection

Six months after initial presentation, our patient tested positive for COVID-19 by RT-PCR. While he was at high risk for complications related to his COVID-19 infection (age >60 years, history of respiratory problems, and presence of a hematologic malignancy), he remained asymptomatic. One month after his COVID-19 diagnosis, he presented with complications associated with transformation to AMML. Though this patient did not experience a cytokine storm characteristic of severe/critical COVID-19 infection, infected cells still secrete type I interferons, which trigger pro-inflammatory mediator and cytokine release, including elevated IL-1β, IL-6, TNFα, and GM-CSF [29]. Interestingly, CMML patients exhibit higher background concentrations of the above-mentioned pro-inflammatory cytokines, and these same pro-inflammatory mediators (IL-1β, IL-6, TNFα) have been reported to promote AML aggressiveness [15]. IL-1β is thought to contribute to leukemic cell expansion in AML by stimulating the proliferation of granulocytes in the marrow and lymphocytes in the spleen [30]. As noted above, TET2 mutations result in upregulated IL-6, and signaling mutations in NRAS/JAK2^V617F result in GM-CSF hypersensitivity [14,16]. Inhibitors of IL-1R, IL-6, and GM-CSF have been found to be promising pharmacotherapy for CMML patients, including those with COVID-19 infection [16,29].

## Conclusions

In conclusion, we present a case report of a patient with myeloproliferative CMML-0 and ulcerative colitis. The presence of TET2 and SRSF2 mutations placed the patient at increased risk of a concurrent SIAD, which was evidenced by the patient’s UC. The patient’s COVID-19 infection, although asymptomatic, may have resulted in increased production of pro-inflammatory mediators and cytokines, which combined with the patient’s CMML, may be a contributing factor in his rapid progression to AMML.
